# Influence of Substrate Location and Temperature Variation on the Growth of ZnO Nanorods Synthesized by Hot Water Treatment

**DOI:** 10.3390/ma17153716

**Published:** 2024-07-27

**Authors:** S. M. Sayem, Ranjitha Kumarapuram Hariharalakshmanan, Emad Badradeen, Shawn E. Bourdo, Tansel Karabacak

**Affiliations:** 1 School of Physical Sciences, University of Arkansas at Little Rock, Little Rock, AR 72204, USA; rhariharalak@ualr.edu (R.K.H.); eobadraddin@ualr.edu (E.B.); txkarabacak@ualr.edu (T.K.); 2 Department of Physics, Applied Physics, and Astronomy, Rensselaer Polytechnic Institute, Troy, NY 12180, USA; 3 Department of Chemistry and Physics, University of Arkansas at Pine Bluff, 1200 N University Dr., Pine Bluff, AR 71601, USA; 4 Center for Integrative Nanotechnology Sciences, University of Arkansas at Little Rock, Little Rock, AR 72204, USA; sxbourdo@ualr.edu

**Keywords:** hot water treatment, metal oxide nanostructure, deionized water, low temperature, hot plate, ZnO, growth, nanorods, metal substrate

## Abstract

Hot water treatment (HWT) is a versatile technique for synthesizing metal oxide nanostructures (MONSTRs) by immersing metal substrates in hot water, typically in glass beakers. The proximity of substrates to the heat source during HWT can influence the temperature of the substrate and subsequently impact MONSTR growth. In our study, zinc (Zn) substrates underwent HWT at the base of a glass beaker in contact with a hot plate and at four different vertical distances from the base. While the set temperature of deionized (DI) water was 75.0 °C, the substrate locations exhibited variations, notably with the base reaching 95.0 °C. Scanning electron microscopy (SEM), energy-dispersive X-ray spectroscopy (EDS), X-ray diffraction (XRD), and Raman spectroscopy showed stoichiometric and crystalline zinc oxide (ZnO) nanorods. ZnO rods on the base, exposed to higher temperatures, displayed greater growth in length and diameter, and higher crystallinity. Nanorods with increasing vertical distances from the base exhibited a logarithmic decrease in length despite identical temperatures, whereas their diameters remained constant. We attribute these findings to crucial HWT growth mechanisms like surface diffusion and “plugging”, influenced by temperature and water flow within the beaker. Our results provide insights for optimizing synthesis parameters to effectively control MONSTR growth through HWT.

## 1. Introduction

Metal oxide nanostructures (MONSTRs) possess a diverse array of electronic and optical properties, which differentiate them from those of their bulk counterparts. These properties are attributed to their high surface-area-to-volume ratio, increased fraction of atoms at the surface of nanomaterials, fewer direct neighbors of the surface atoms, and the quantum confinement effect [1,2,3,4]. Zinc oxide (ZnO) is a promising material with a wide band gap (3.37 eV), large exciton binding energy (60 meV at room temperature), and high electron mobility (205–300 cm^2^ V^−1^ s^−1^) [5,6,7]. Recent research has focused on the study of nanostructures of n-type metal oxides, such as ZnO, owing to their potential applications in ultraviolet lasers, optoelectronics, catalysis, energy storage and conversion, field emission, sensors, and transistors [8,9,10,11,12]. ZnO’s properties are significantly impacted by factors such as its crystal shape (spherical, conical, spiral, cylindrical, etc.), as well as nanostructure size, orientation, and density [13,14]. Over the past years, various methods have been employed to produce ZnO nanostructures with various properties. Conventional methods, such as e-beam evaporation [15], sputtering [16,17,18], chemical vapor deposition (CVD) [19,20], pulsed laser ablation (PLA) [21,22], and atomic layer deposition (ALD) [23], have been used to synthesize ZnO nanostructures with various properties. However, these methods have several limitations, including the requirement of high temperature, high vacuum, and expensive specialized equipment. Alternative techniques, such as chemical bath deposition (CBD) [24]; hydrothermal [25,26], solvothermal [27,28], and electrochemical deposition [29]; and precipitation methods [30] have been explored to address these issues. Nevertheless, these alternative methods also have drawbacks, including the use of surfactants, toxic additives, and multistep preparation processes. Therefore, a low-temperature, atmospheric-pressure, low-cost, scalable, simple, and eco-friendly process is essential for producing ZnO nanostructures.

Hot water treatment (HWT) is a solution-based method that involves immersing a metal plate in pure deionized water at a constant low temperature (less than 100 °C) to create MONSTRs on a metal surface without the need for surfactants or chemical additives [31,32,33]. This approach is both simple and cost-effective, requiring only a metal plate, deionized (DI) water, and a hot plate. The use of pure DI water minimizes the risks of contamination from impurities during HWT and the toxicity of wastewater to biological organisms after HWT. Various types of elemental metals, alloys, and compounds have been used to fabricate MONSTRs with diverse morphologies using HWT [31]. Furthermore, ZnO nanostructures, including nanowires, nanorods, and nanosheets, have been fabricated using HWT [34,35].

Lately, the rapid advancement of functional nanostructured surfaces has sparked the interest of researchers in the growth of wurtzite ZnO structures on zinc surfaces through hot water treatment. Many of these studies have concentrated on the diverse applications and performance tests of metal oxides produced by HWT [36,37,38,39], while only a few have addressed the underlying growth mechanism of this novel synthesis process. According to Tan et al., surface diffusion is the dominant process during HWT [40]. In a more detailed study, Saadi et al. observed that “plugging” could also be a major growth mechanism during MONSTR growth by HWT [31]. Briefly, a sequence of chemical reactions occurs at the water–metal interface during HWT, forming metal oxide molecules. Subsequently, these molecules undergo a multistep process called the plugging process, which involves their release from the metal surface into the water, migration in the water, and redeposition onto the metal surface to form MONSTRs. Through surface diffusion, the rough surfaces of the formed MONSTRs gradually transform into smooth surfaces. Furthermore, various parameters such as treatment time, dissolved oxygen, water purity, and water flow pattern can influence the growth of MOSNTRs by HWT. The temperature of the water environment during HWT is a critical parameter for controlling the growth of MONSTRs [41,42,43]. In previous studies that utilized HWT, there was not enough clarity regarding how the synthesis temperature was controlled. Some studies have reported the set temperature on the hot plate, but the actual temperature of the water inside the glass beaker might be different [43]. In other works, a temperature-control rod was used; however, the metal substrate was placed at the bottom of a glass beaker [12,31,38]. In such cases, the temperature-control rod maintained the water temperature, but the surface of the hot plate and the base of the glass beaker in contact with it could be at a much higher temperature. These temperature variations could lead to variations in the growth of MONSTRs. Therefore, a comprehensive study is necessary to understand the growth mechanism of MONSTRs in response to temperature fluctuations during HWT.

In this work, we synthesized ZnO nanorods on zinc (Zn) plates utilizing HWT by placing the Zn plates at the base of a glass beaker and at various vertical distances from it. The temperatures at distinct locations were measured under similar HWT conditions (set temperature, DI water volume, and treatment time). Subsequently, the average lengths and diameters of the nanorods were calculated, and their morphology, composition, and structure were analyzed. Additionally, a discussion of the possible relationship between the temperature and water flow pattern on the growth of the nanorods is presented, accompanied by a detailed potential growth mechanism. This study aims to provide insights to the research community about some of the critical experimental parameters and growth mechanisms to be considered for the precise control of the growth of MONSTRs by not only the HWT method but also other solution-based synthesis methods using similar experimental designs.

## 2. Materials and Methods

For this study, Zn plates of 99.9% purity, 0.51 mm thickness, and of 35.31 mm × 35.31 mm area were purchased from ESPI. Ultrapure DI water of 18.2 MΩ.cm resistivity was generated in the laboratory using a combination of the Micropure ST and Mili-Q Academic systems eBay (San Jose, CA, USA). P3000 sandpaper, Al_2_O_3_ abrasives of 60 grit size, and K-type thermocouple probes were purchased from Amazon.com, Seattle, CA, USA. Acetone and isopropanol were purchased from Fisher Scientific (Waltham, MA, USA). The hot plate used in this study was a Corning PC-420D VWR (Radnor, PA, USA) with a temperature-control rod.

**Figure 1:**
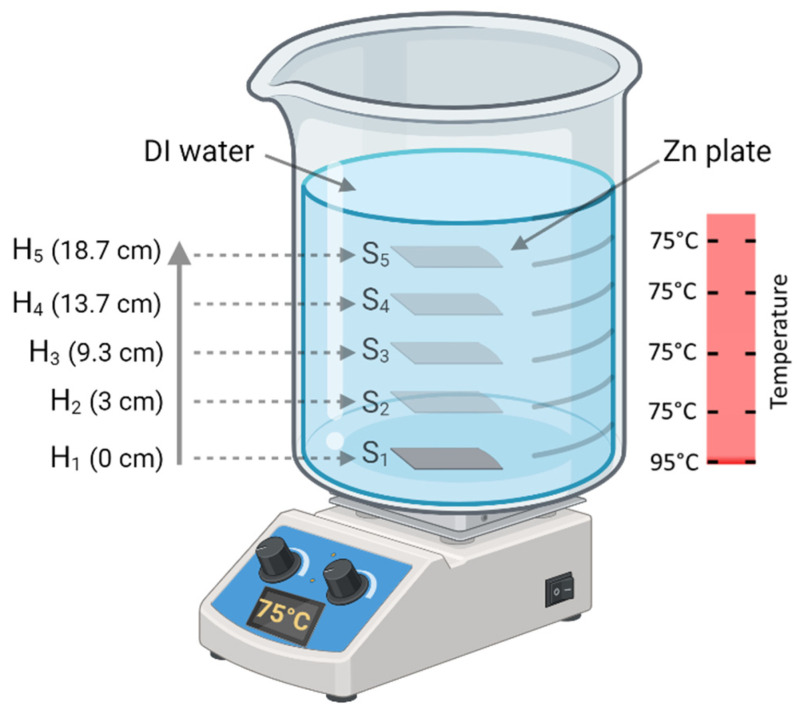
Schematic diagram of hot water treatment (HWT) indicating distinct positions of zinc (Zn) substrates and temperature inside the beaker.

Five Zn plates were polished using sandpaper to eliminate organic contaminants and native oxide. Next, these plates were subjected to sandblasting with aluminum oxide (Al_2_O_3_) abrasives at a pressure of 20 psi in a closed container to introduce micro-roughness on their surfaces. The plates were then cleaned by ultrasonication in acetone, isopropanol, and DI water for 10 min each. For the HWT experiment (Figure 1), 3500 mL of DI water inside a glass beaker was maintained at a constant temperature of 75.0 °C using a temperature-control rod. One cleaned Zn plate was immersed in hot DI water and placed at the bottom of the beaker for 1 h. This process was repeated four times using different Zn plates at different vertical heights inside the beaker, using a custom-made Teflon setup (see Supplementary Figure S1). The Zn plate samples were named accordingly, sample S_1_ at location H_1_, sample S_2_ at location H_2_, etc. (Figure 1). After the treatment, the plates were air-dried at room temperature for 1 h and transferred for characterization. All characterizations were performed on the side of the substrate facing upward during HWT, except for S_5_, in which scanning electron microscopy (SEM) images were taken on both sides (upward and downward faces) of the substrate (described in the Section 3). The sample plates were dipped in liquid nitrogen and then broken into pieces before obtaining the cross-sectional electron microscopy images of the ZnO nanorods. The process was successful without any obstacles, in contrast to the HWT aluminum oxide (Al_2_O_3_) grown on Al in our previous study [44]. This is believed to be because there is no notable difference between the thermal expansion coefficients of Zn and ZnO [45,46]. The temperature of DI water at different Zn plate locations within the beaker was measured using a k-type thermocouple system. The temperature measurements were performed beginning at the base and proceeding in the horizontal and vertical directions, including all the locations depicted in Figure 1. The thermocouple tip was maintained at its exact location until the readings stabilized. The recorded temperature at the base surface of the beaker was 95.0 ± 1.6 °C, whereas the temperature at all other locations was 75.0 ± 0.5 °C (Figure 1).

**Figure 2:**
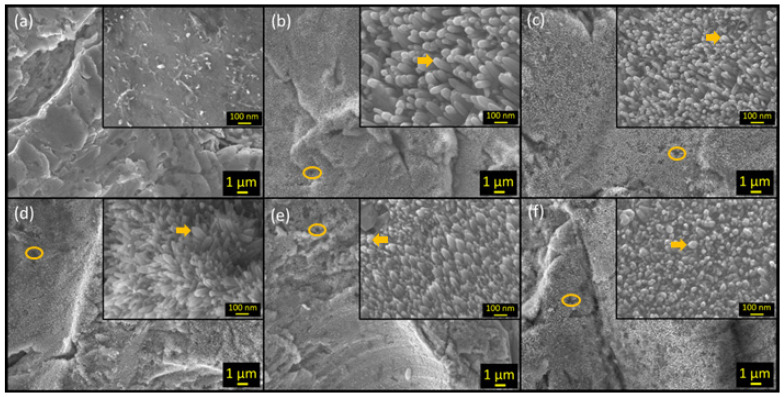
Scanning electron microscopy (SEM) images of (**a**) Zn plate before HWT (**b**–**f**) ZnO nanorods after HWT on the Zn plate samples S_1_–S_5_, respectively. Insets show high magnification of the nanorods.

**Figure 3:**
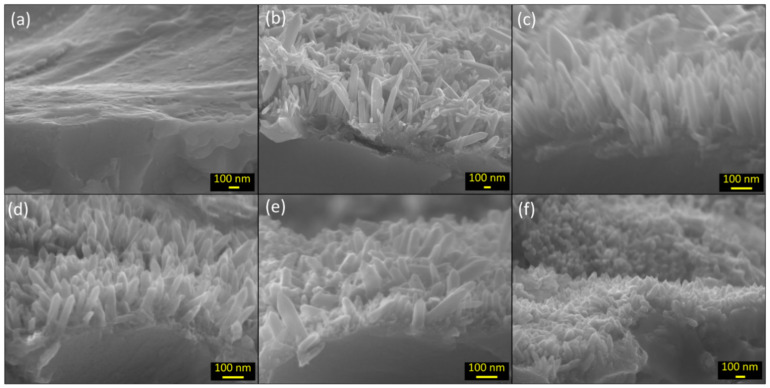
Cross-sectional SEM images of (**a**) zinc plate before HWT and (**b**–**f**) ZnO nanowires grown after HWT on the Zn plate samples S_1_–S_5_, respectively.

SEM (JOEL JSM-7000F, Tokyo, Japan) equipped with field emission (FE) and operated at 15 kV along with energy-dispersive X-ray spectroscopy (EDS) (EDAX Element, Pleasanton, CA, USA; operated at 5 kV) were used to analyze the morphology and map the chemical composition of the Zn plate surfaces. The SEM images in Figure 2 were captured at a magnification of 5000×, with insets at a magnification of 100,000×. The magnification for Figure 3 varied for each image: (a) 50,000×, (b) 33,000×, (c–e) 100,000×, and (f) 45,000×. The EDS results shown in Supplementary Figure S2 were obtained at a magnification of 75,000×. The crystal structure of the ZnO nanorods was analyzed by X-ray diffraction (XRD, Rigaku Miniflex 600, Tokyo, Japan) with Cu Kα radiation (*λ* = 1.5418 Å) and Raman scattering spectra (“LabRam HR800”, Horiba Jobin Yvon (Piscataway, NJ, USA)) at a laser wavelength of 514 nm.

## 3. Results and Discussion

The SEM images presented in Figure 2 depict the top views of the polished and sandblasted Zn plates before and after HWT treatment. Figure 2a reveals the micro-roughness of the Zn plate (S_c_) prior to HWT treatment, where no nanofeatures were visible. Following only one hour of HWT, ZnO nanorods were observed on the surfaces of the Zn plates (S_1_–S_5_), as illustrated in Figure 2b–f. The ZnO nanorods exhibited the most uniform and dense growth when the substrate was in contact with the base of the beaker (S_1_), as demonstrated in Figure 2b. In Figure 2c–f, noticeable gaps between the nanofeatures can be observed (marked by yellow ovals), which may indicate that the nanorods grown on these samples exhibited lower density (i.e., number of nanorods per area) and uniformity than S_1_. The high-magnification SEM images in each figure provide a closer look at the nanorods. It is evident that the surfaces of all nanorods are smooth and possess hexagonal facets (indicated by yellow arrows).

**Figure 4:**
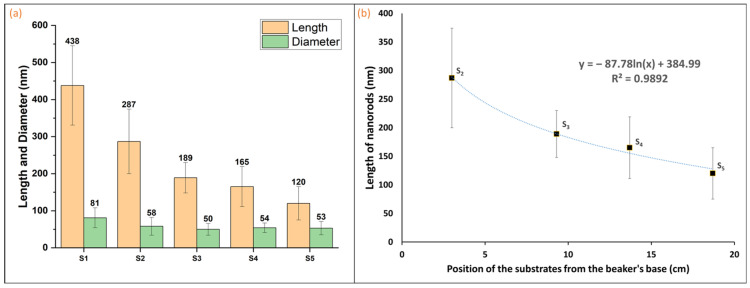
(**a**) Bar diagram comparing the average lengths and diameters of the ZnO nanorods grown on all the Zn plates S_1_–S_5_ and (**b**) ZnO nanorod length vs. position of the Zn plates S_2_–S_5_.

The SEM images in Figure 3 show the cross-sections of all the samples before (Figure 3a) and after (Figure 3b–f) HWT. The lengths and diameters of the ZnO nanorods were measured using these images, and the mean values and their standard deviations are presented in Figure 4a. The ZnO nanorods grown on S_1_ were the longest, approximately 438 nm in length and 81 nm in diameter. A progressive decrease in the length of the nanorods was observed from S_2_ (~287 nm) to S_5_ (~120 nm) (Figure 4b), whereas the diameter varied less relatively. It is clear from the SEM images that the nanorods in all the samples had hexagonal pointed tips.

Additionally, EDS was used to analyze the chemical composition of the as-grown ZnO nanorods on top of the Zn plates after HWT (see Supplementary Figure S2). In all the specimens, the characteristic peaks found were only zinc and oxygen, with an approximately 1:1 ratio of Zn to O atoms, as shown in Supplementary Table S1. Moreover, the weight percentage of Zn to O was approximately 80 to 20, similar to that of the bulk ZnO weight composition.

**Figure 5:**
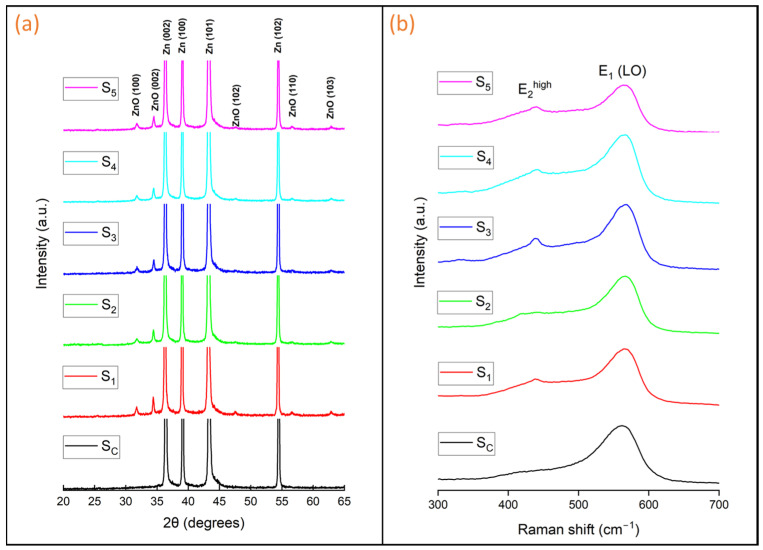
(**a**) X-ray diffraction (XRD) patterns, and (**b**) Raman spectra of the Zn plates before and after HWT.

Figure 5a displays the XRD patterns of the Zn plates before and after HWT. The control sample (S_C_) exhibited diffraction patterns at 2θ = 36.2°, 39.0°, 43.2°, and 54.3°, corresponding to the (002), (100), (101), and (102) planes of Zn, respectively [crystallography open database entry 9008522]. All the other samples (S_1_ to S_5_) showed diffraction peaks at 2θ = 31.7°, 34.4°, 47.7°, 57.1°, and 63.1°, corresponding to the (100), (002), (102), (110), and (103) planes of the wurtzite crystal structure of ZnO ([40], crystallography open database entry 1011258). The presence of the predominant ZnO (002) peak suggests that the ZnO nanostructures in all samples were mainly aligned along the c-axis [40]. Additionally, the ZnO peaks of S_1_ were more intense and sharper than those of the other samples, confirming that the ZnO nanorods produced at the bottom of the beaker were more crystalline and vertically oriented than those produced at different positions inside the beaker during HWT. The individual ZnO nanorods are believed to be single crystals as indicated by their faceted shapes observed in the SEM images. However, the XRD profiles revealed peaks corresponding to different crystal planes of ZnO, which is most likely due to the random orientation of the ZnO nanorods with respect to the substrate plane.

Additionally, Debye–Scherrer’s formula (Equation (1)) was used to determine the approximate average crystallite size of the ZnO nanorods.

(1) $$D=K\lambda /\left(\beta \cos\theta \right)$$

where *λ* = X-ray wavelength (1.54060 Å), *K* = Scherrer constant (0.9), *β* = full width at half-maximum (FWHM) of the diffraction peak (in radians), and *θ* = Bragg’s diffraction angle. The calculated approximate crystallite sizes from S_1_ to S_5_ are 30.7 nm, 29.3 nm, 28.7 nm, 25.9 nm, and 25.5 nm, respectively. Given that the relationship between β and D is inverse, the approximate crystallite values indicate that S_1_ exhibits the most pronounced ZnO peak, whereas S_5_ exhibits the least pronounced peak.

The Raman scattering spectra of the Zn plates before and after 1 h of HWT are depicted in Figure 5b. The crystal structure of ZnO is hexagonal wurtzite, which belongs to the P6_3_mc space group with Raman-active modes comprising A_1_ + 2E_2_ + E_1_ [47]. The peak at 438 cm^−1^, which corresponds to the nonpolar E_2_^high^ phonon mode, indicates the presence of the wurtzite hexagonal phase of ZnO [40]. The polar E_1_ (LO) mode at 568 cm^−1^ is related to the oxygen vacancies in ZnO nanorods [34]. The more pronounced and intense 438 cm^−1^ peak at S_1_ further supports the XRD results, indicating that the ZnO nanorods formed at S_1_ (i.e., at the base of the beaker) were more crystalline than the others (Supplementary Figure S3). Conversely, the intensity of the 568 cm^−1^ peak gradually decreased from S_1_ to S_5_, suggesting a decrease in oxygen deficiency. This could be because the samples closer to the surface of the water had better access to dissolved oxygen, leading to the observed change in intensity. Furthermore, we quantified the ratio of the intensity of E_1_ (LO) to that of E_2_^high^ for S_1_ and S_5_. The ratio for S_1_ is 1.57, whereas that for S_5_ is 1.70. These results suggest that the ZnO nanorods fabricated at the bottom of the beaker were more crystalline and exhibited lower oxygen deficiency than the ZnO nanorods produced near the water surface. For the control sample, the E_1_ (LO) peak observed was more pronounced than some of the hot water-treated samples, which could be attributed to the build-up of amorphous native oxide on the bare zinc surface. Although XRD is not as sensitive in identifying native oxide films on metal surfaces, Raman spectroscopy is a more effective method for this purpose. Interestingly, no E_2_^high^ peak was detected in the control sample, and only an E_1_ (LO) peak was observed, suggesting that native ZnO may be present in an amorphous form.

The more significant increase in the growth of the ZnO nanorods on S_1_ can be attributed to the differences in the measured temperature inside the beaker. S_1_ was positioned at the bottom of the beaker, where the temperature was the highest, specifically at 95.0 °C. The nanorods grown on this specific sample were both the longest and the widest. An increased temperature should also result in an increase in the release of Zn ions into the water, leading to increased formation of the ZnO molecules. Additionally, even though our thermocouples were unable to measure it, within a short distance from the surface of the S_1_ substrate, the temperature of the water could still be higher than 75.0 °C [26]. This could lead to faster migration and/or redeposition of previously released ZnO molecules onto the S_1_ substrate. The accelerated rates of release, migration, and redeposition of ZnO molecules suggest that the plugging mechanism may have significantly contributed to the increased growth of the nanorod length observed at S_1_. The larger diameter of S_1_ can be explained by the island (or Volmer–Weber) growth mode and shadowing effect [48,49]. In the early stages of HWT, it is anticipated that the growth rate of nanostructures will be more rapid because of the higher concentration of dissolved oxygen in the water. As the deposition rate increased, a larger number of atoms were present on the metal surface, resulting in a higher likelihood of these adatoms sticking together. This leads to the formation of ZnO islands and rods with small diameters [50]. However, the rate of surface diffusion during nanoparticle growth is significantly influenced by temperature, which can affect the surface mobility of redeposited oxide molecules [50]. The higher temperature of the S_1_ substrate may have played a crucial role in determining the likelihood of adatoms possessing sufficient energy to move from the top surface of the growing island to its side surfaces. Consequently, this could have led to an expansion in the diameter of the islands. Moreover, it is hypothesized that the preferential redeposition of the released ZnO molecules on the hill tops and tips of the nanorods compared to the valleys on the micro-roughened Zn plate surface may result in the “shadowing effect” phenomenon [31,50]. For S_1_, surface diffusion is expected to be stronger and compete with the shadowing effect. A higher surface diffusion can force some of the redeposited ZnO molecules to move along the sides of the rods (instead of just the tips for other samples because of the shadowing effect and limited surface diffusion) and increase their diameter. This, in addition to the high surface diffusion of the initial islands of ZnO, can create nanorod bases of larger diameters. Thus, the increased diameter of the nanorods on S_1_ compared with the other samples may be attributed mainly to the higher temperature of this substrate, which was conducted from the base of the beaker. Additionally, the initial ZnO nanorods may possess rough surfaces owing to the random nature of plugging [31]. Consequently, the ZnO molecules that are redeposited can diffuse over the surface, resulting in the smooth, faceted appearance of the nanorods, as depicted in the SEM images.

It is anticipated that the rate of surface diffusion and plugging will be comparatively lower for the remaining substrates than for S_1_ because of their lower temperature (75.0 °C). Consequently, the average length and diameter of the nanorods grown on these substrates (S_2_–S_5_) were generally smaller than those grown on S_1_. Nonetheless, the rates of surface diffusion and plugging should be largely equivalent for these substrates because of the uniformity of the measured water temperature (75.0 °C). Accordingly, the anticipated expansion of nanorods on these surfaces should be comparable. Our results demonstrate that the average diameters of the nanorods are nearly identical; however, the lengths of the nanorods from S_2_ to S_5_ exhibited a logarithmic decline (R^2^ = 0.9892 and 0.9862 for logarithmic and exponential fits, respectively). This declining trend from S_2_ to S_5_ could be attributed to the plugging mechanism rather than the surface diffusion mechanism, as the temperature remained nearly constant. As mentioned in the introduction, the plugging mechanism involves the formation, release, migration, and redeposition of ZnO molecules. For samples S_2_ through S_5_, the ZnO formation and release are believed to be identical since all of them are at the same temperature. However, the migration and redeposition steps could have been affected by a change in the water flow pattern within the beaker. During HWT, owing to convection, hot water from the bottom of the beaker should rise upward, and cold water from the top should move downward, following a circular pattern. It is highly possible that the ZnO molecules released from the S_1_ substrate did not encounter any impediments following the same path as the water molecules because the substrate was situated near the origin of the flow, and the ZnO molecules could have a higher chance of redepositing back on the substrate due to the circulating flow pattern. Nevertheless, the other substrates were situated directly in the course of the convection flow, which could have led to a greater deposition of the released ZnO molecules on the bottom face of the substrates (side facing downward) than on their top face (facing upward). To test this hypothesis, we conducted an experiment similar to that of S_5_, but examined the bottom face of the Zn plate. The average length and diameter of the resulting nanorods were 155 nm and 51 nm, respectively, while the nanorods on the front side had an average length of 120 nm and a diameter of 53 nm. These findings imply that more deposition of ZnO molecules may have occurred on the bottom-facing side of the substrates in samples S_2_–S_5_ than on the top side. Furthermore, the distance between the substrate and water surface decreased more significantly for sample S_5_ than for any other sample. As a result, the likelihood of ZnO molecules flowing from the rear of the substrate to the front and being redeposited diminished for sample S_5_ compared to the other samples. This could be a contributing factor to the reduction in the length of the nanorods from S_2_ to S_5,_ as shown in Figure 4. In addition, the relatively consistent growth of ZnO nanorods on the S_1_ sample discussed above could potentially be attributed to the faster rate of plugging and the shadowing effect occurring near this substrate compared to the other substrates. This is demonstrated by the SEM images, which show a less uniform formation of nanostructures on the other substrates. Further research is necessary to elucidate the underlying mechanism of the ZnO nanorod growth.

## 4. Conclusions

HWT is a flexible strategy for growing nanostructures on metallic substrates. This study examined the growth of ZnO nanorods at various locations within a beaker containing DI water using the HWT method. Additionally, this study explored the connection between a range of synthesis parameters, such as temperature, dissolved oxygen, and water flow pattern, and the formation of nanorods through different growth mechanisms, including plugging, surface diffusion, and shadowing effects. A comparative morphological analysis was performed on the ZnO nanorods grown using SEM. Additionally, XRD and Raman spectroscopy results indicated that the nanorods exhibited strong crystallinity. The temperature of the beaker surface in contact with the hot plate was significantly higher (95.0 °C) than the set temperature of the water (75.0 °C). The ZnO nanorods grown on the Zn substrate (S_1_) in the comparatively high-temperature region exhibited a more significant growth in both length and diameter than the substrates (S_2_–S_5_) placed in the intended temperature (75.0 °C) region within the beaker water. This marked increase in growth is attributed to the higher temperature, which leads to an increased rate of plugging and surface diffusion mechanisms. In addition, the shadowing effect has the potential to further promote the growth of elongated nanorods. However, the nanorods in the lower temperature location showed a logarithmic decrease in length but no change in diameter, even though the temperature was constant at all those locations. The effect of water flow patterns at different substrate locations may be responsible for this trend in nanorod growth. Our study showed that the actual temperature of the Zn substrates placed at the base of the glass beaker differed significantly from the set water temperature at different substrate locations within the beaker during HWT. Nonetheless, the growth of metal oxide nanostructures can be controlled by optimizing the growth conditions and placing the substrates in suitable locations inside the beaker. This research can also provide insights into the growth mechanisms of other solution-based synthesis methods that utilize similar experimental arrangements.

## Data Availability

The original contributions presented in the study are included in the article/Supplementary Material, further inquiries can be directed to the corresponding author.

## Supplementary Materials

The following supporting information can be downloaded at: https://www.mdpi.com/article/10.3390/ma17153716/s1, Figure S1: Custom-made Teflon setup to hold Zn plates at a fixed position during HWT; Figure S2: EDS mapping of samples S_1_–S_5_ after HWT; Figure S3: Overlap of the Raman spectra of the Zn plates before and after HWT; Table S1: Weight and atomic percentage of all the samples by EDS.

## References

[1] Joudeh N., Linke D. (2022). Nanoparticle classification, physicochemical properties, characterization, and applications: A comprehensive review for biologists. J. Nanobiotechnol..

[2] Nizami M.Z.I., Xu V.W., Yin I.X., Yu O.Y., Chu C.-H. (2021). Metal and metal oxide nanoparticles in caries prevention: A review. Nanomaterials.

[3] Li L., Wang P., Shao Q., Huang X. (2020). Metallic nanostructures with low dimensionality for electrochemical water splitting. Chem. Soc. Rev..

[4] Maciulis V., Ramanaviciene A., Plikusiene I. (2022). Recent advances in synthesis and application of metal oxide nanostructures in chemical sensors and biosensors. Nanomaterials.

[5] Jabeen M., Asharaf M.W., Tayyaba S., Ali N., Kumar R.V., Alrobei H. (2021). Growth of zinc oxide nanowires by equimolar solution technique on conducting substrates used for optical applications. Dig. J. Nanomater. Biostruct..

[6] Sodeinde K.O., Olusanya S.O., Lawal O.S., Sriariyanun M., Adediran A.A. (2022). Enhanced adsorptional-photocatalytic degradation of chloramphenicol by reduced graphene oxide-zinc oxide nanocomposite. Sci. Rep..

[7] Yun S., Guo T., Li Y., Gao X., Huang A., Kang L. (2020). Well-ordered vertically aligned zno nanorods arrays for high-performance perovskite solar cells. Mater. Res. Bull..

[8] Wei X., Deng Y., Hu X., Zhao J., Wei H., Yang Z., Han G. (2023). Biomass derived fibrous porous carbon loaded zinc oxide nanoparticles as high-performance anode materials for lithium ion batteries. J. Energy Storage.

[9] Chen Y.-C., Tu Y.-H., Chen L.-W., Lai Y.-H., Tsai M.-F., Lin Y.-X., Lai H.-C., Chiang C.-Y., Liu H.-J., Pan H.-C. (2021). Fabrication of large-scale high-mobility flexible transparent zinc oxide single crystal wafers. ACS Appl. Mater. Interfaces.

[10] Mostafa A.M., Mwafy E.A., Awwad N.S., Ibrahium H.A. (2021). Linear and nonlinear optical studies of ag/zn/zno nanocomposite thin film prepared by pulsed laser deposition technique. Radiat. Phys. Chem..

[11] Bah A., Lim K.Y., Wei F., Khursheed A., Sow C.H. (2019). Fluorescence invigoration in carbon-incorporated zinc oxide nanowires from passage of field emission electrons. Sci. Rep..

[12] Hariharalakshmanan R.K., Martinez J., Ergul-Yilmaz B., Karabacak T. (2023). Suspension of zno nanostructures synthesized by hot water treatment for photocatalytic wastewater treatment. Water Air Soil Pollut..

[13] Yin J., Gao F., Wei C., Lu Q. (2014). Water amount dependence on morphologies and properties of zno nanostructures in double-solvent system. Sci. Rep..

[14] Zhang L., Jeem M., Okamoto K., Watanabe S. (2018). Photochemistry and the role of light during the submerged photosynthesis of zinc oxide nanorods. Sci. Rep..

[15] Giri P.K., Panchal C.J., Bhattacharyya S., Kumari S., Singh D.K., Kheraj V.A., Shah N.M., Vakil P.D., Patel K.J., Desai M.S. (2007). Studies on zinc oxide nanorods grown by electron beam evaporation technique. Synth. React. Inorg. Metal-Org. Nano-Met. Chem..

[16] Deng Z., Tian Y., Yin X., Rui Q., Liu H., Luo Y. (2008). Physical vapor deposited zinc oxide nanoparticles for direct electron transfer of superoxide dismutase. Electrochem. Commun..

[17] Varnagiris S., Urbonavicius M., Tuckute S., Lelis M. (2021). Formation of zn-rich zno films with improved bulk and surface characteristics by approach of magnetron sputtering technique. Thin Solid Films.

[18] Puthiyottil H., Thankamani P.R., Saji K.J. (2023). Exploring the effects of substrate and substrate temperature on the properties of radio frequency magnetron sputtered zno thin films. Mater. Today Commun..

[19] Jia F., Shih Y.-L., Pui D.Y.H., Li Z.-Y., Tsai C.-J. (2021). Generation of zno nanoparticles by chemical vapor synthesis using quenching air. J. Nanopart. Res..

[20] Sazanova T.S., Mochalov L.A., Logunov A.A., Kudryashov M.A., Fukina D.G., Vshivtsev M.A., Prokhorov I.O., Yunin P.A., Smorodin K.A., Atlaskin A.A. (2022). Influence of temperature parameters on morphological characteristics of plasma deposited zinc oxide nanoparticles. Nanomaterials.

[21] Rashid S.N., Aadim K.A., Jasim A.S., Hamad A.M. (2022). Synthesized zinc nanoparticles via pulsed laser ablation: Characterization and antibacterial activity. Karbala Int. J. Mod. Sci..

[22] Mohamed T., Farhan A., Ahmed H., Ashour M., Mamdouh S., Schuch R. (2022). Nonlinear optical properties of zinc oxide nanoparticle colloids prepared by pulsed laser ablation in distilled water. Nanomaterials.

[23] Seo G.H., Yun D.J., Lee W.H., Yoon S.M. (2017). Atomic-layer-deposition-assisted zno nanoparticles for oxide charge-trap memory thin-film transistors. Nanotechnology.

[24] Nandanapalli K.R., Mudusu D. (2018). Surface passivated zinc oxide (zno) nanorods by atomic layer deposition of ultrathin zno layers for energy device applications. ACS Appl. Nano Mater..

[25] ElFaham M.M., Mostafa A.M., Mwafy E.A. (2021). The effect of reaction temperature on structural, optical and electrical properties of tunable zno nanoparticles synthesized by hydrothermal method. J. Phys. Chem. Solids.

[26] Chakraborty A., Orsini A., Kar J.P., Gatta F., Khan U., Falconi C. (2022). Ultra-efficient thermo-convective solution-growth of vertically aligned zno nanowires. Nano Energy.

[27] Aga K.W., Efa M.T., Beyene T.T. (2022). Effects of sulfur doping and temperature on the energy bandgap of zno nanoparticles and their antibacterial activities. ACS Omega.

[28] Hu Y., Sun L., Liu Z., Liu T. (2023). Controlled solvothermal synthesis of zno nanoparticles using non-destructive mg-based channel templates for enhanced photocatalytic performance. Mater. Chem. Phys..

[29] Quang N.X., Luyen N.T., Hue N.T., Nhung P.T.T., Khi N.T., Lam V.D., Le A.-T., Thuy N.T.T., Huy T.Q. (2023). Formation and antibacterial activity of heterogeneous zinc oxide nanoparticles greenly synthesized by the electrochemical method under microwave treatment. Colloids Surf. A Physicochem. Eng. Asp..

[30] Rajan S., Venugopal A., Kozhikkalathil H., Valappil S., Kale M., Mann M., Ahuja P., Munjal S. Synthesis of zno nanoparticles by precipitation method: Characterizations and applications in decipherment of latent fingerprints. Mater. Today Proc..

[31] Saadi N.S., Hassan L.B., Karabacak T. (2017). Metal oxide nanostructures by a simple hot water treatment. Sci. Rep..

[32] Hu Y., Li R., Zhang X., Zhu Y., Nie H.-Y. (2020). Aluminium films roughened by hot water treatment and derivatized by fluoroalkyl phosphonic acid: Wettability studies. Surf. Eng..

[33] Khedir K.R., Saifaldeen Z.S., Demirkan T.M., Al-Hilo A.A., Brozak M.P., Karabacak T. (2015). Robust superamphiphobic nanoscale copper sheet surfaces produced by a simple and environmentally friendly technique. Adv. Eng. Mater..

[34] Hariharalakshmanan R.K., Watanabe F., Karabacak T. (2022). In situ growth and uv photocatalytic effect of zno nanostructures on a zn plate immersed in methylene blue. Catalysts.

[35] Tan W.K., Razak K.A., Lockman Z., Kawamura G., Muto H., Matsuda A. (2013). Optical properties of two-dimensional zno nanosheets formed by hot-water treatment of zn foils. Solid State Commun..

[36] Pelicano C.M., Yanagi H. (2019). Ph-controlled surface engineering of nanostructured zno films generated via a sustainable low-temperature h2o oxidation process. Appl. Surf. Sci..

[37] Pelicano C.M.O., Raifuku I., Ishikawa Y., Uraoka Y., Yanagi H. (2020). Hierarchical core–shell heterostructure of h 2 o-oxidized zno nanorod@ mg-doped zno nanoparticle for solar cell applications. Mater. Adv..

[38] Hariharalakshmanan R.K., Saadi N.S., Ergul-Yilmaz B., Al-Mayalee K.H., Karabacak T. (2020). Zinc oxide nanostructures synthesized by a simple hot water treatment method for photocatalytic degradation of organic pollutants in water. MRS Adv..

[39] Hammer B.I., Hariharalakshmanan R.K., Sayem S., Haque S., Karabacak T. (2024). Durability of metal oxide nanostructures synthesized by hot water treatment. MRS Commun..

[40] Tan W.K., Razak K.A., Lockman Z., Kawamura G., Muto H., Matsuda A. (2013). Formation of highly crystallized zno nanostructures by hot-water treatment of etched zn foils. Mater. Lett..

[41] Pelicano C.M., Yanagi H. (2017). Efficient solid-state perovskite solar cells based on nanostructured zinc oxide designed by strategic low temperature water oxidation. J. Mater. Chem. C.

[42] Aslan M.M. (2012). Nanostructuring of alumina optical waveguides by hot water treatment for tuning sensor output. Thin Solid Films.

[43] Basher M.K., Riyadh S.M.S., Hossain M.K., Hassan M., Akand A.R., Zumahi S.M.A.-A., Matin A., Das N., Nur-E-Alam M. (2023). Development of zinc-oxide nanorods on chemically etched zinc plates suitable for high-efficiency photovoltaics solar cells. Opt. Quantum Electron..

[44] Smith Q., Burnett K., Saadi N., Haque S., Badradeen E., Sayem S., Ali N., Bush J., Karabacak T. (2023). Enhancing antibacterial property of aluminum foil by nanostructuring its surface through a steam treatment. MRS Adv..

[45] Ibach H. (1969). Thermal expansion of silicon and zinc oxide (ii). Phys. Status Solidi (B).

[46] Callister W.D., Rethwisch D.G., Blicblau A., Bruggeman K., Cortie M., Long J., Hart J., Marceau R., Mitchell R. (2007). Materials Science and Engineering: An Introduction.

[47] Huang Q., Liu J. (2013). Facile and clean solution synthesis of large-scale zno nanorods assisted with aliquat 336. J. Chem..

[48] Park S.-H., Seo S.-Y., Kim S.-H., Han S.-W. (2006). Surface roughness and strain effects on zno nanorod growth. Appl. Phys. Lett..

[49] Karabacak T. (2011). Thin-film growth dynamics with shadowing and re-emission effects. J. Nanophotonics.

[50] Yussuf N.A.M. (2022). Growth and Application of Titanium Nanorods with Branches Using Physical Vapor Deposition. Ph.D. Thesis.

