# Supplementary material for: Influence of Substrate Location and Temperature Variation on the Growth of ZnO Nanorods Synthesized by Hot Water Treatment
Source: Materials (Basel). 2024 Jul 27;17(15):3716. doi: 10.3390/ma17153716 (PMC11312918; doi:10.3390/ma17153716)
Supplement: Supplementary file 1 [file materials-17-03716-s001.zip › materials-3098470-supplementary.pdf]

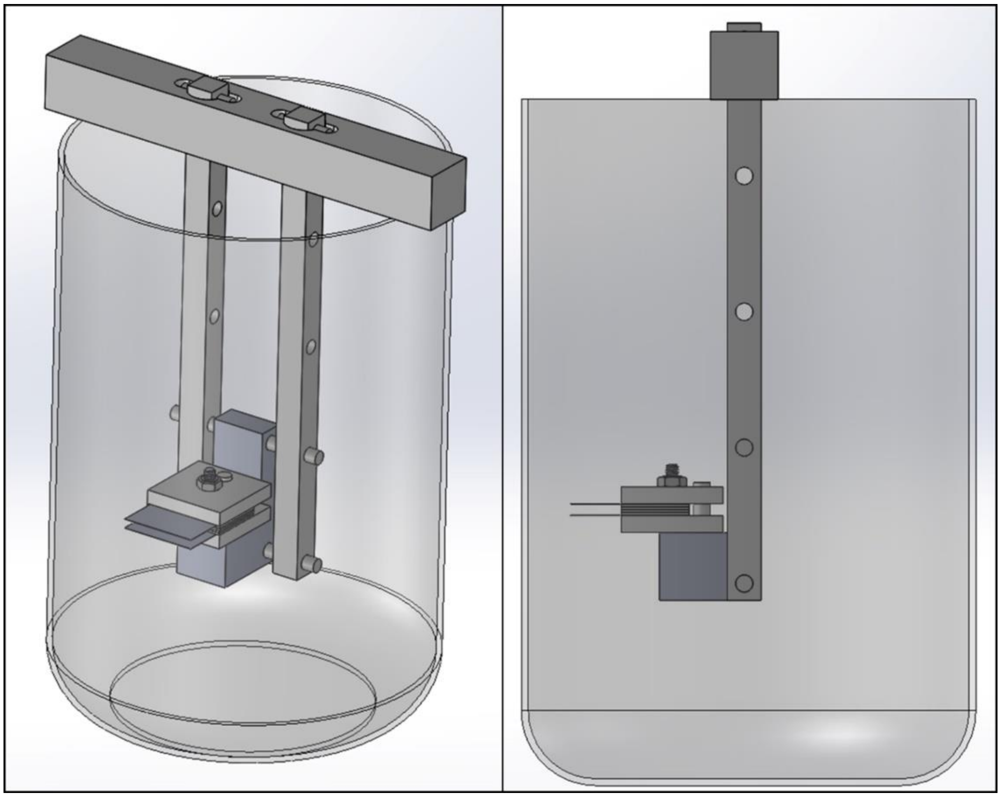

Figure S1. *Custom made Teflon setup to hold Zn plates at a fixed position during HWT.*

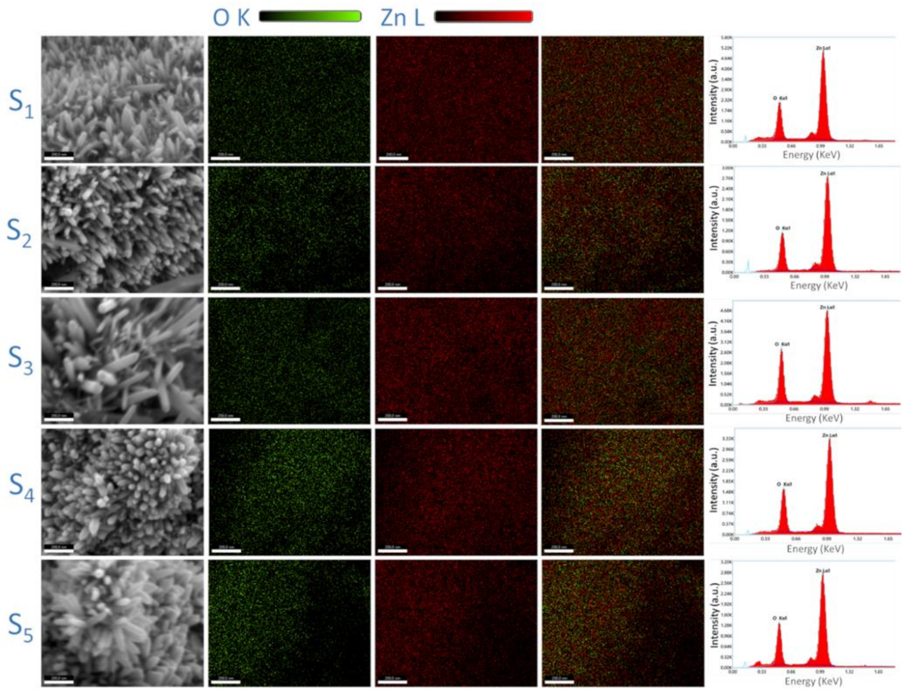

Figure S2. *EDS mapping of samples S_1 – S_5 after HWT.*

Table S1. *Weight and atomic percentage of all the samples by EDS.*

Sample	Element	Weight %	Atomic %	Net int.	Error %
**S1**	O K	19.8	50.3	87.2	7.3
	Zn L	80.2	49.7	168.0	6.3
**S2**	O K	18.9	48.7	45.2	7.7
	Zn L	81.1	51.3	93.1	6.5
**S3**	O K	23.7	55.9	82.4	7.3
	Zn L	76.3	44.1	124.8	6.4
**S4**	O K	21.0	52.1	62.5	7.5
	Zn L	79.0	47.9	111.6	6.4
**S5**	O K	20.5	51.3	52.1	7.6
	Zn L	79.5	48.7	95.9	6.5

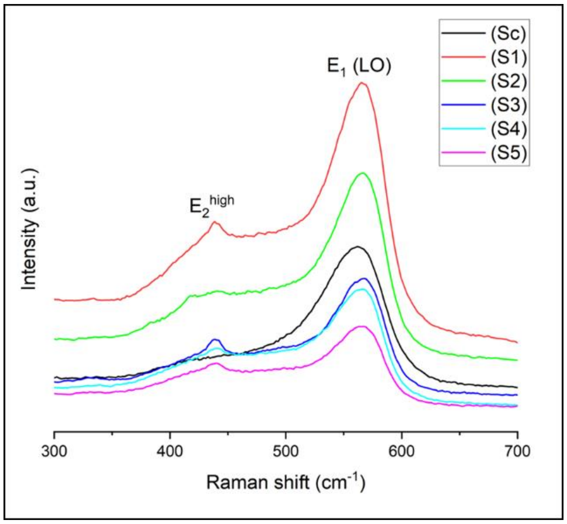

Figure S3. *Overlap of the Raman spectra of the Zn plates before and after HWT.*
